# XBP1- IGFBP3 Signaling Pathway Promotes NSCLC Invasion and Metastasis

**DOI:** 10.3389/fonc.2021.654995

**Published:** 2021-05-18

**Authors:** Qingxi Luo, Wenwen Shi, Bo Dou, Jun Wang, Wei Peng, Xianyu Liu, Deze Zhao, Faqing Tang, Yingfang Wu, Xizhe Li, Jiajia Li, Siqi Wen, Chunfang Zhang, Chaojun Duan

**Affiliations:** ^1^ Department of Thoracic Surgery, Xiangya Hospital, Central South University, Changsha, China; ^2^ Hunan Engineering Research Center for Pulmonary Nodules Precise Diagnosis & Treatment Xiangya Hospital, Central South University, Changsha, China; ^3^ Department of Oncology, Hunan Provincial People’s Hospital/The First Affiliated Hospital of Hunan Normal University, Changsha, China; ^4^ Hunan Key Laboratory of Oncotarget Gene, Department of Clinical Laboratory, Hunan Cancer Hospital & The Affiliated Cancer Hospital of Xiangya School of Medicine, Central South University, Changsha, China; ^5^ Centre of Stomatology, Xiangya Hospital, Central South University, Changsha, China; ^6^ Institute of Medical Sciences, Xiangya Lung Cancer Center, Xiangya Hospital, Central South University, Changsha, China; ^7^ National Clinical Research Center for Geriatric Disorders, Xiangya Hospital, Central South University, Changsha, China

**Keywords:** NSCLC, XBP1, IGFBP3, invasion, metastasis

## Abstract

Lung cancer is the most frequently diagnosed cancer and the main cause of cancer death in the world. X-box binding protein 1 (XBP1), which is an important transcription factor involved in regulating the unfolded protein response (UPR) during endoplasmic reticulum (ER) stress, might act as a potent oncogenic protein in the processes of tumorigenesis, tumor proliferation and metastasis in various cancers. However, the clinical significance and pathological role of XBP1 in non-small cell lung cancer (NSCLC) remains unknown. In this study, we investigated the expression of XBP1s protein in the 104 NSCLC tumor tissues and matched adjacent normal lung tissues (ANLT) by Immunohistochemical (IHC), and we found overexpressed XBP1s protein was associated with NSCLC TNM stages, lymph node metastasis and poor prognosis. The further gain-and loss-of-function experiments indicated overexpression of XBP1s protein promoted cell invasion, migration and metastasis both *in vitro* and *in vivo*. Further study showed XBP1s protein could upregulate insulin-like growth factor binding protein-3 (IGFBP3) expression, and regulated NSCLC cells invasion and metastasis by regulating IGFBP3. Taken together, XBP1s protein is markedly overexpressed in NSCLC and serves as an oncogene that play a critical role in NSCLC tumorigenesis and development. Importantly, XBP1s protein might not only be a potential biomarker for metastasis and prognosis but also a potential therapeutic target in NSCLC.

## Introduction

According to the Global cancer statistics 2020 (1), in both sexes combined, lung cancer has become the most frequently diagnosed cancer in the world and the main cause of cancer death (2). And in 2019, the Cancer Statistics from the American Cancer Society indicated lung cancer remains the leading cause of cancer death in the United States (3). According to histopathology, lung cancer is mainly divided into small cell lung cancer and non-small cell lung cancer (NSCLC) and NSCLC comprises approximately 85% of all lung cancers. Early stage (stage I or II) lung cancer patients are difficult to diagnose because of lack of obvious symptoms, most portion of lung cancer patients are diagnosed with locally advanced or metastasis disease (stage III or IV), at the time surgical resection is no longer possible (4). Still now, conventional chemotherapy and radiation therapy are still the most important treatment for NSCLC patients. However, tumor metastasis and recurrence are the main reasons for the poor prognosis of patients with NSCLC, the specific mechanism of NSCLC development and metastasis is still unclear. Recently, a large scale molecular mechanisms studies of NSCLC’ proliferation and metastasis have made a significant progress in diagnosis, prognostic and therapeutic strategies of non-small cell lung cancer (5).

X-box binding protein 1 (XBP1) is an important transcription factor involved in regulating the unfolded protein response (UPR) during endoplasmic reticulum (ER) stress, it has been found to be widely expressed in various tumors and is closely related to the tumorigenesis and progression (6, 7). In addition, XBP1 acts as an important role in protection against oxidative stress *via* ROS signaling pathway in several cancers (8, 9). There are two forms, including spliced XBP1 (XBP1s) and un-spliced XBP1 (XBP1u), and XBP1s is formed by XBP1u, which is spliced and removed 26 nucleotide intron by activated RNase (10). Inositol requiring enzyme 1α (IRE1α)-XBP1 signaling pathway is the most conserved branch of the UPR pathways and plays a crucial role in maintaining ER homeostasis. Accumulating evidence demonstrated that IRE1α-XBP1 pathway plays a critical role in various cancers, IRE1α-XBP1 pathway was found playing an important role in cell proliferation, tumor progression and invasion in colorectal carcinoma (11), melanoma (12), hepatocellular carcinoma (13) and prostate cancer (14). Moreover, XBP1 is widely expressed in various cancers (15–18). Previous study demonstrated that XBP1s mRNA levels are highly expressed in lung cancer (19), however, the biological role and molecular mechanisms of XBP1 in NSCLC remain unknown. In this study, we reveal that overexpression of XBP1 promotes NSCLC tumorigenesis, invasion and metastasis by regulating IGFBP3 expression.

## Materials and Methods

### Tissue Samples and Clinic Pathological

A total of 104 pairs of NSCLC and matched ANLTs (adjacent non‐tumor lung tissues) were collected for this study. All tissues we collected were from patients who underwent a Surgical lobectomy at Xiangya Hospital, CSU. The specimens were collected with the patients informed. The Patients were followed up for 5 years to obtain prognostic information. All the collected samples were evaluated by histopathologist. The clinicopathologic staging of patients with NSCLC was determined according to the TNM grading criteria for stage 8 lung cancer. The clinic pathological characteristics of the 104 samples were summarized in **Table 1**. We kept the collected NSCLC and matched ANLTs in -80°C refrigerator. 

**Table 1:** Correlation analysis of clinicopathological features between XBP1 and NSCLC patients.

Characteristics	Number	XBP1 expression		P value
		High(n)	Low(n)	
Age(years)				
<60	55	39	16	0.21
≥60	49	29	20	
Gender				
Male	78	53	25	0.341
Female	26	15	11	
Smoking history				
No	37	24	13	0.934
Yes	67	44	23	
Pathological type				
Adenocarcinoma	67	32	35	0.479
Squamous cell carcinoma	37	15	22	
Differentiation				
Well	24	18	6	0.007
Moderate	56	40	16	
Poor	24	9	15	
Tumor invasion				
T1	31	20	11	0.989
T2	44	29	15	
T3	21	13	8	
T4	8	5	3	
Lymph node metastasis				
Yes	39	30	9	0.009
No	65	36	29	
TNM stage				
I	33	17	16	0.034
II	35	21	14	
III+IV	36	29	7	

### Cell lines and Cell Culture

NSCLC cell lines A549, H1299, Calu-1, PC-9, SK-MES were purchased from the Type Culture Collection (ATCC, Manassas, VA) in the United States. These cells were cultured in a 10% fetal bovine serum (FBS) and penicillin/streptomycin RPMI 1640 and placed in a 5% humidified CO2 incubator at 37°C. All cell lines were identified by short tandem repeats (STR) profiling prior to use. The cells were used within 10 passages.

### Viruses and Transduction

The XBP1 lentivirus were synthesized and purified by Genechem (Shanghai, China), and si-XBP1 plasmid (the information of siRNA sequence is in the **Table 2**) were purchased from Ribobio (Guangzhou, China). The cell transfection methods were performed according to the manufacturer’s protocol, after lentivirus transduction, A549,H1299 and Calu-1 cells were treated with 2 µg/ml puromycin to obtaining stable expressed cell lines. Cells were harvested for further analysis of XBP1 expression at 48h after transfection.

**Table 2:** siRNA sequence.

si XBP1	GGTATTGACTCTTCAGATT
si IGFBP3	GCTACAGCATGCAGAGCAA

### Quantitative Real-Time PCR

Total RNA was extracted from cells and tissue samples using TRIzol reagent. For measuring the expression level of IGFBP3 mRNA, 1 μg of RNA was reverse-transcribed using a Primerscript RT reagent kit with gDNA Eraser and quantitative real-time PCR was performed using SYBR Green PCR Master Mixture. The information of IGFBP3 and internal control Actin primers as follow:

IGFBP3: forward, 5’- GCGCCAGGAAATGCTAGTGA-3’;
reverse, 5’- GGGGTGGAACTTGGGATCAG-3’;
β-Actin: forward,5’- CCTGTACGCCAACACAGTGC-3’;
reverse, 5’-ATACTCCTGCTTGCTGATCC-3’;

### Xenografted Tumor Model and H&E Staining

We conducted a BALB/C nude mouse lung colonization model to investigate the invasion effect of XBP1 on NSCLC progression. Four-week-old Male BALB/c‐nu mice were purchased from the Laboratory Animal Department of Central South University (Changsha, China). The mice we got were randomly divided into two groups (n = 5 per group). We next injected A549^XBP1-OE^ cells or A549^XBP1-NC^ cells (1 × 10^6^ cells each mouse) *via* lateral tail veins into each groups. Two months later we killed the mice and gathered the lungs for H&E staining.

### Western Blotting

The total protein extracted from cells were separated by 10% sodium dodecyl sulfate polyacrylamide gel electrophoresis (SDS–PAGE), and then transferred to a PVDF membrane. The membrane was next incubated with 5% skim milk for 2 h at 20-25°C. After being incubated with primary antibodies at 4°C for 10h and then with rabbit or mouse secondary antibodies at 20-25°C for 1h. Signals were detected using chemiluminescence reagents. The primary antibodies used were anti‐XBP1 (1:1000, ab37152, abcam), anti‐IGFBP3. (1:1000 10189-2-AP,PTGCN), anti‐Tubulin antibody (1:5000. D110016, Sangon Biotech, Shanghai, China).

### Immunofluorescent Staining

We used anti‐XBP1 (1:100, ab37152, abcam) and anti‐IGFBP3. (1:100 10189-2-AP, PTGCN) to perform IHC analysis on NSCLC tissue microarrays.

### *In Vitro* Migration and Invasion Assay

For wound healing assay, we used a 10-μL pipette tip to scratch fused monolayers grown in six‐well plates. We obtained images by an inverted phase contrast microscope 24 h later. And then we measured the width of a wound using Image Pro Plus 6.0. we seeded cells in serum‐free medium in transwell chamber (BD, USA) without matrigel in 24-well plates for the migration assay, or covered with matrigel (BD, USA) for the invasion assay. The bottom chamber was added 600 µl 1640 medium containing 10% FBS. 48h latter, we washed the upper chambers with PBS and then air-dried it. We dyed it with crystal violet and counted from six random fields under inverted microscope.

### Mass Spectrometry

The protein extracted from A549 transfected with LV-XBP1 was treated with dithiothreitol (DTT) and iodoacetamide, and then determined using the Bradford method. 200ug protein was added to 4 times the volume of acetone then centrifuged and dried. For trypsin digestion, trypsin was added three times every 6 hours at 37°C. Digested peptides were then treated with 0.1% formic acid in water, washed in 0.1% formic acid (FA) in acetonitrile. The mixture was lyophilized and then reconstituted in 20 μL 0.1% FA. and the mixture was subjected to LC-MS/MS with an LTQ Orbitrap Velos mass spectrometer coupled to an Ultimate RSLCnano LC system (Thermo Scientific, Waltham, MA, USA). Raw data were processed using the Proteome Discoverer v.1.4 and matched to the Swiss-Prot human database. Four sets of LC-MS/MS data were standardized and filtered (proteins ≥2 peptides and an averaged area ratio-fold change ≥1.50 or ≤0.67 were considered differentially expressed proteins).

### Co-Immunoprecipitation

The cell lysate for co-immunoprecipitation, the supernatant was incubated with corresponding antibody overnight (2 μg antibody per 500 μg protein sample) and with protein A+G magnetic beads (HY-K0202, Med Chem Express) for 4 h at 4°C. HA-specific magnetic beads (HY-K0201, Med Chem Express) were used for HA-fusion protein. Antibodies used in immunoprecipitation are described below: XBP1 (ab37152, abcam), IGFBP3 (ab193910, abcam). The magnetic beads were isolated by the magnetic racket and washed by phosphate buffered saline supplemented with 0.5% Triton-100 (PBST). Samples were eluted with 0.1M glycine pH 3.0 adjusted to pH 7.5 with Tris buffer and run on an SDS-PAGE. After SDS-PAGE electrophoresis, proteins in the gels were detected by silver staining and followed by in-gel trypsin digestion and MS analysis as previously described by us.

### Statistical Data Analysis

All statistical analyses in this study were carried out using SPSS 22.0 statistical software. Values were shown as mean ± SD, and 2‐tailed paired Student’s t‐test was used for comparing the difference between groups unless otherwise stated. The chi‐square test was used to analyze the protein expression levels and clinic pathologic parameters. Spearman’s rank correlation determined the association between XBP1 protein and IGFBP3 protein. The log‐rank test was used to compared the patient survival curves. *P* < 0.05 was considered significant.

## Result

### Clinical Significance of XBP1s Protein Overexpression in NSCLC

**Figure 1:**
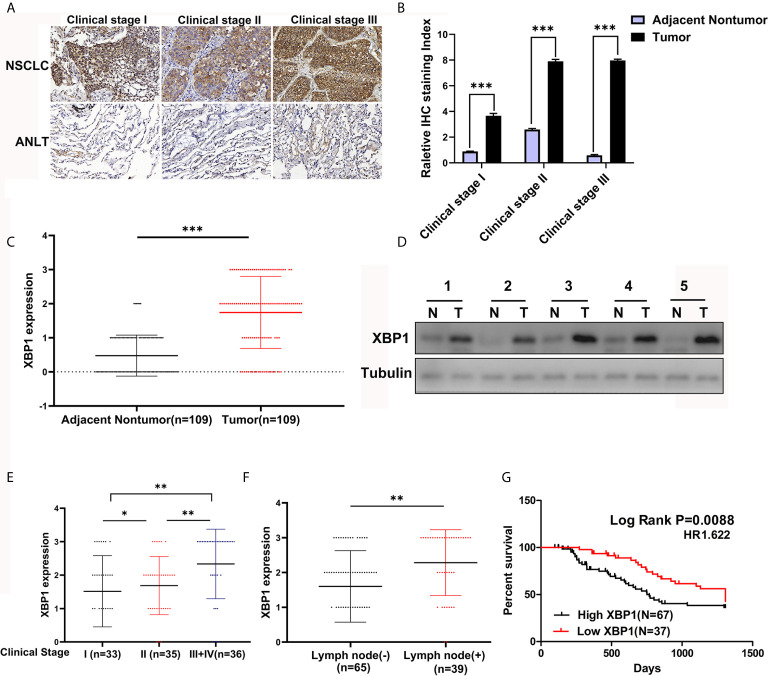
XBP1 is upregulated in NSCLC tumor tissues and associated with poor prognosis. **(A–C)** Representative immunohistochemically stained images of human NSCLC tumor tissues and the matched adjacent normal lung tissues using anti-XBP1 antibody, and relative protein expression level of XBP1 in NSCLC tumor tissues compared to the matched adjacent normal lung tissues from 104 patients. **(D)** Western blot analysis of XBP1 expression in 5 paired human NSCLC tumor tissues (T) compared to the matched adjacent normal lung tissues (N). **(E, F)** advanced TNM stage or Lymph node metastasis NSCLC tumors had higher XBP1 expression level. **(G)** Kaplan-Meier overall survival curves for NSCLC patients indicated the high XBP1 expression level is correlated with worse overall survival rates. Data were represented as the mean ± SEM of three independent experiments. *P < 0.05, **P < 0.01, ***P < 0.001.

According to the previous study, XBP1 is comprehensively overexpressed in cancer cells (14, 17, 20, 21). In this study, we measured the expression level of XBP1 in 104 NSCLC tumor tissues and matched adjacent normal lung tissues (ANLTs) using immunohistochemical (IHC). As shown **Figures 1A–C** shows that the expression of XBP1s protein in NSCLC tumor tissues was significantly elevated compared to the ANLTs, especially in advanced NSCLC samples. Meanwhile, the same results were obtained by western blot assay (**Figure 1D**). In addition, we collected and compared clinical data of 104 NSCLC patients, **Table 1** show that overexpressed XBP1 protein was associated with TNM stages (*P*<0.001) and lymph node metastasis (*P*<0.005). XBP1s protein expression was higher in NSCLC with lymph node metastasis and advanced stage (**Figures 1E, F**). The clinicopathological analysis and Log-rank test suggest that overexpressed XBP1 may be related with NSCLC progression and prognosis. We used Kaplan-Meier analysis to confirm the relationship between XBP1 expression and 3-year overall survival (OS). The results revealed that the 3-year OS for the low XBP1 expression group was higher than the high expression group (**Figure 1G**).

### XBP1 Is Associated With the Invasion and Metastasis of NSCLC *In Vitro*

**Figure 2:**
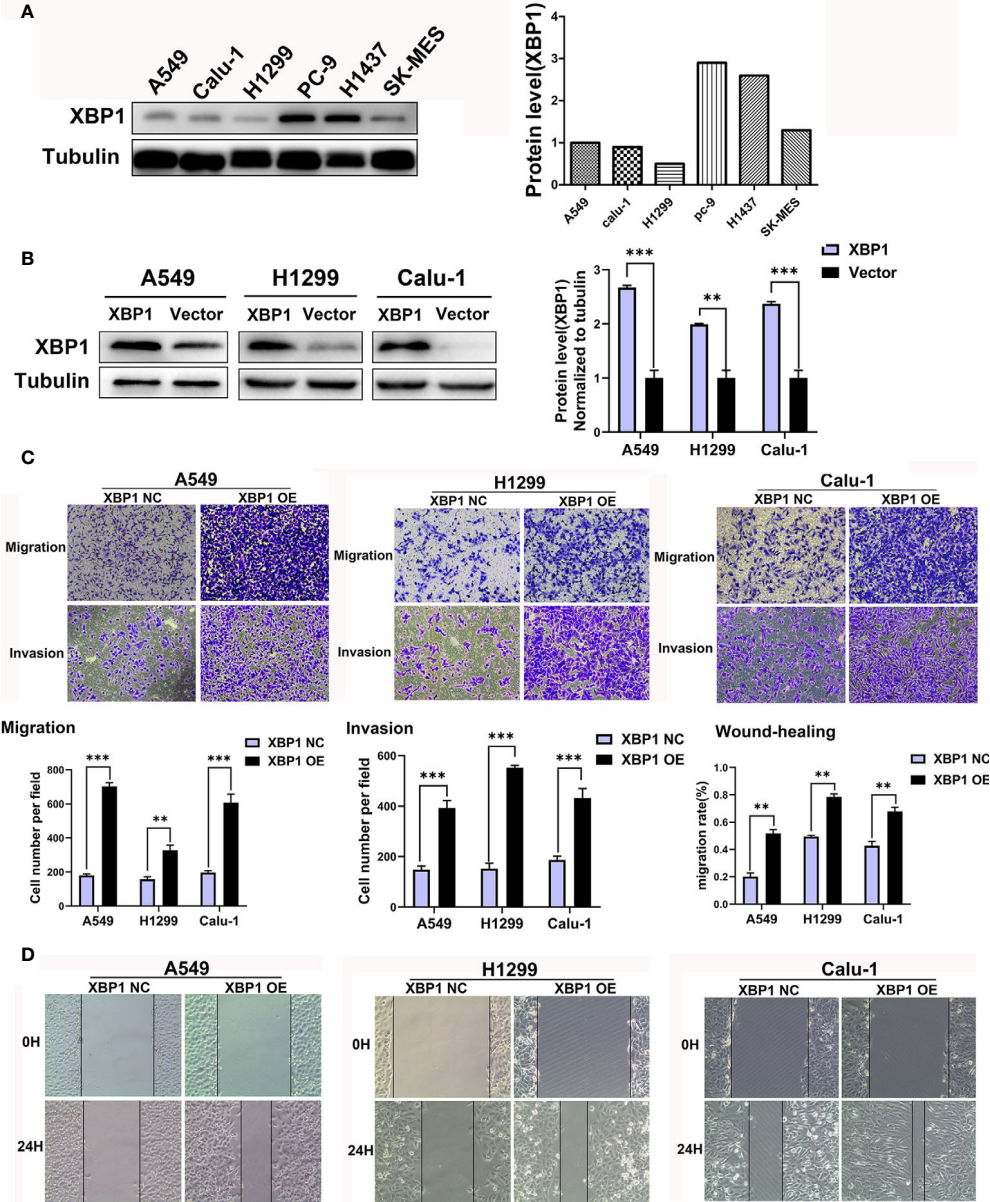
XBP1 overexpression promotes lung cancer cells’ migration and invasion. **(A)** Western blot analysis of XBP1 expression in NSCLC cell lines. The highest expression of XBP1 protein occurs in PC-9 cells. **(B)** Western blot showed the expression of XBP1 in A549, H1299 and Calu-1 cells after transfected with XBP1-overexpression or negative control. **(C)** Transwell migration and invasion assays of A549, H1299 and Calu-1 cells transfected with XBP1 overexpression and negative control. **(D)** Wound-healing assay of A549, H1299 and Calu-1 cells transfected with XBP1 overexpression and negative control. Data were represented as the mean ± SEM of three independent experiments. **P < 0.01, ***P < 0.001.

**Figure 3:**
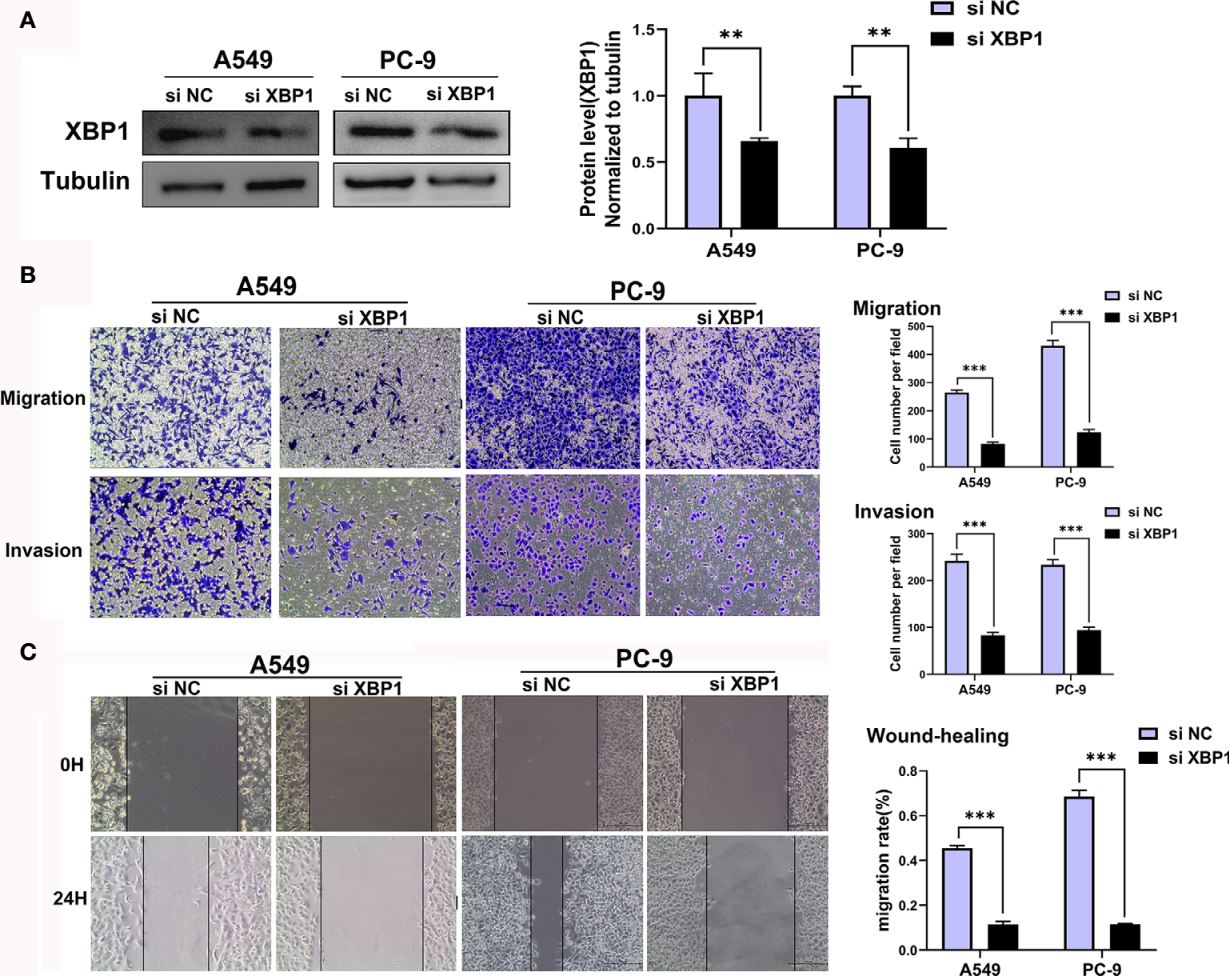
Downregulated XBP1 expression inhibits lung cancer cells’ migration and invasion. **(A)** Western blot assay showed the expression of XBP1 in A549 and PC-9 transfected with si-XBP1 and negative control. **(B)** Transwell migration and invasion assays of A549 and PC-9 cells transfected with si-XBP1 and negative control. **(C)** Wound-healing assay of A549 and PC-9 cells transfected with si-XBP1 and negative control. Data were represented as the mean ± SEM of three independent experiments. **P < 0.01, ***P < 0.001.

We further explored the expression of XBP1 protein in lung cancer cell lines and found that A549, H1299 and calu-1 exhibited low expression of XBP1 and PC-9 exhibited the highest expression (**Figure 2A**). A549, H1299, calu-1 and PC-9 were selected for further studies. After transfection, XBP1 overexpression cell lines was higher than the matching XBP1 control cell lines (**Figure 2B**) and XBP1 siRNA effectively suppressed the expression of XBP1 in A549 and PC-9 (**Figure 3A**). According to the clinicopathological analysis, the overexpression of XBP1s protein was significantly associated with NSCLC tumor stage and lymph node metastasis, and then we investigated the role of XBP1 in the progress of migration, invasion and metastasis of NSCLC. The wound-healing assay (**Figure 2D**) and the transwell invasion assay and metastasis assay (**Figure 2C**) showed that the overexpression of XBP1 promoted lung cancer cell migration, invasion and metastasis. And further experiment indicated XBP1 siRNA significantly inhibits lung cancer migration, invasion and metastasis in A549^si-XBP1^ and PC-9^si-XBP1^ compare with A549^si-NC^ and PC-9^si-NC^ (**Figures 3B, C**). Overall, these findings indicate that XBP1 is associated with the invasion and metastasis of NSCLC *in vitro*.

### XBP1 Promotes NSCLC Proliferation and Metastasis *In Vivo*

**Figure 4:**
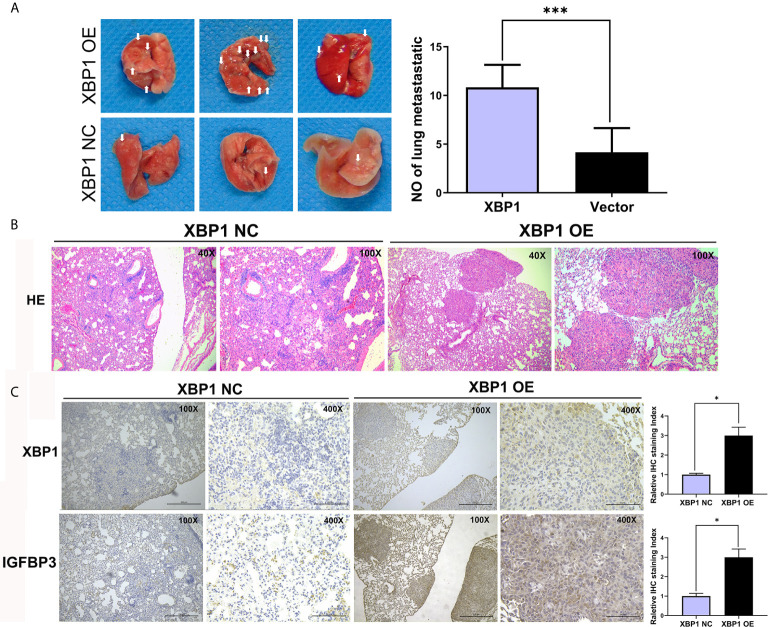
XBP1 overexpression promotes proliferation and metastasis *in vivo*. **(A)** Relative photographs of gross lungs with arrows pointing to lung surface tumor nodules from the i.v. metastasis assay. **(B)** H&E stained section of lung metastasis nodules. With original magnification: X4, X10 are shown. **(C)** Relative immunohistochemically stained images of lung tissues using anti-XBP1 and anti-IGFBP3. Data were represented as the mean ± SEM of three independent experiments. *P < 0.05, ***P < 0.001.

Because we have found that the overexpression of XBP1 correlates with NSCLC progression and metastasis *in vitro*, the further experiments were used to explore the role of XBP1 in lung cancer cells’ proliferation and metastasis *in vivo*. The A549 variants (A549 ^XBP1-OE^ and A549 ^XBP1-NC^) were injected into the tail vein of mouse model. A549 ^XBP1-OE^ and A549 ^XBP1-NC^ were injected according to previously described methods (22, 23). Compared with the control group, A549 ^XBP1-OE^ group showed more and bigger lung metastatic nodules (**Figure 4A**) and H&E stains confirmed more metastasis to the lung (**Figure 4B**). Moreover, IHC using XBP1 and IGFBP3 show corresponding alternation (**Figure 4C**). The results indicate that XBP1 promotes NSCLC proliferation and metastasis *in vivo*.

### XBP1 Interacts With IGFBP3 and Upregulates the Protein Level of IGFBP3

**Figure 5:**
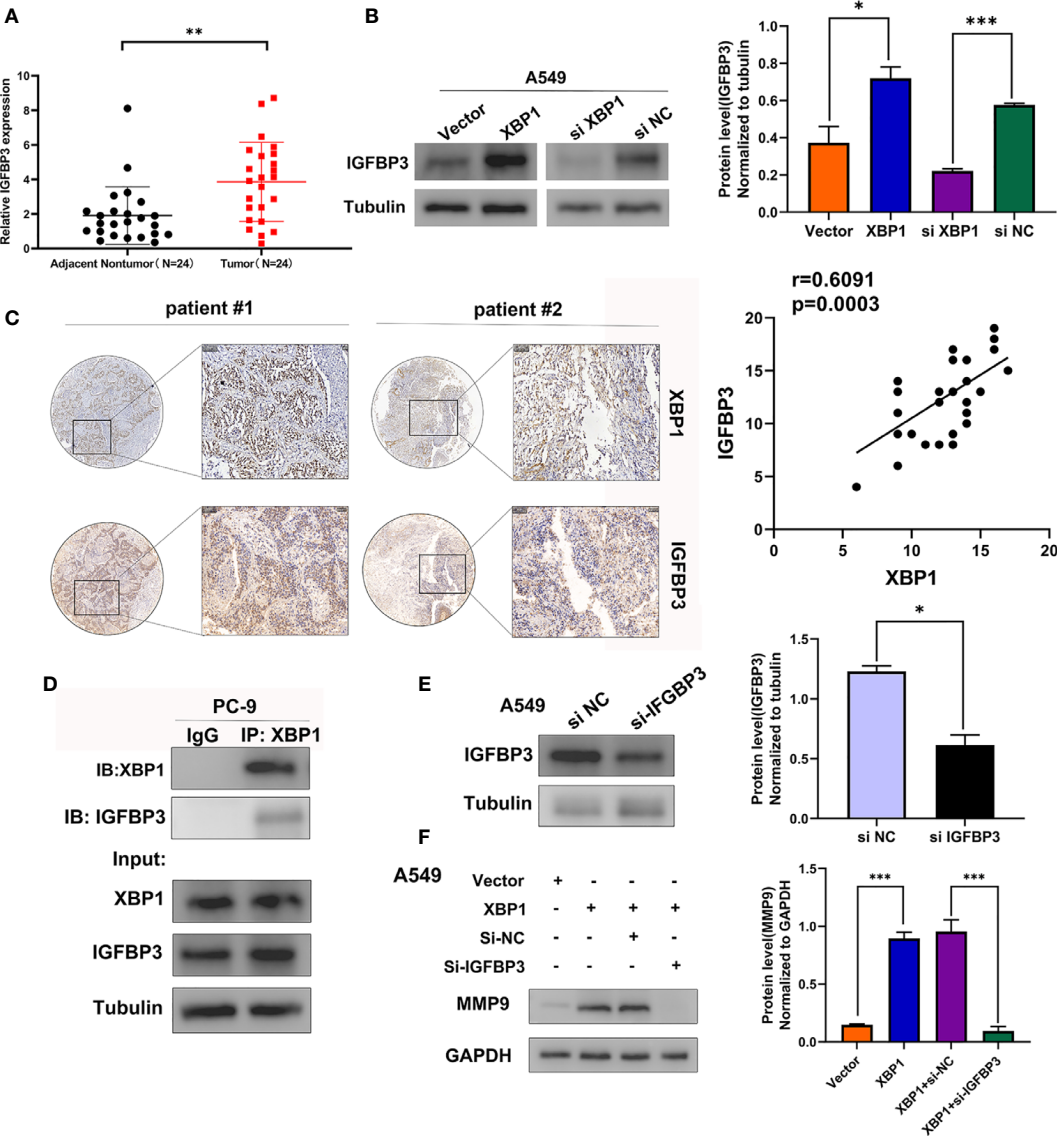
XBP1 interacts with IGFBP3 and upregulates the protein level of IGFBP3. **(A)** Representative statistics analysis of IGFBP3 mRNA expression levels in 24 NSCLC tumor tissues and adjacent nontumor lung tissues using qRT-PCR. **(B)** Expression of IGFBP3 in transfected with XBP1 and negative control cells, si-XBP1 and negative control cells as detected by western blot. **(C)** Representative immunohistochemically stained images of NSCLC tissues using the anti-XBP1 and anti-IGFBP3 antibodies. Areas in the black squares are magnified in the right slide panels. **(D)** Exogenous interaction between XBP1 and IGFBP3 in PC-9 cells. **(E)** Expression of IGFBP3 in transfected with si-IGFBP3 and negative control cells as detected by western blot. **(F)** Expression of MMP-9 in transfected negative control, XBP1, both XBP1 and si-IGFBP1 or both XBP1 and si-NC. Data were represented as the mean ± SEM of three independent experiments. *P < 0.05, **P < 0.01, ***P < 0.001.

To identify the XBP1 interacting proteins, we performed LC-MS/MS analysis in A549 ^XBP1-OE^ and A549 ^XBP1-NC^ cells, the result showed 917 differentially expressed genes (including 875 upregulated genes and 42 downregulated genes)(**Supplementary Table 1**) in A549 ^XBP1-OE^ and A549 ^XBP1-NC^ cells group. Of the 875 upregulated genes, 80 genes were found only express in the A549 ^XBP1-OE^ cells and 39 genes only in the A549 ^XBP1-NC^ cells (**Supplementary Table 2**). And then, we selected 7 cancer-related genes (DNAJB2, USP47, FKBP2, SEC24D, PYCR1,MANF and IGFBP3) in 80 upregulated genes (Unique Peptides>2 Σ# PSMs>10) and evaluated the expression of the selected 7 genes in NSCLC by Oncomine (https://www.oncomine.org/) (**Supplementary Figure S1**), we found that only IGFBP3 was highly expressed in NSCLC and the expression of the selected 7 genes in NSCLC tumor tissues (**Figure 5A**). We used western blot to examine the IGFBP3 protein expression in XBP1 overexpression cells compared to negative control cells, and found that the expression of XBP1 effectively promotes the expression of IGFBP3 (**Figure 5B**). Further IHC analysis showed IGFBP3 is also highly expressed in NSCLC tumor tissues and is positively correlated with the expression of XBP1 expression (**Figure 5C**). In PC-9 cell line, immunoprecipitated using anti-XBP1antibody followed by WB using anti-IGFBP3 antibody showed the combination between XBP1 and IGFBP3 (**Figure 5D**). Taken together, these findings suggest that XBP1 interacts with IGFBP3 and upregulates IGFBP3 protein level.

### XBP1 Regulates NSCLC Cells Invasion and Metastasis by Regulating IGFBP3/MMP-9 Axis

**Figure 6:**
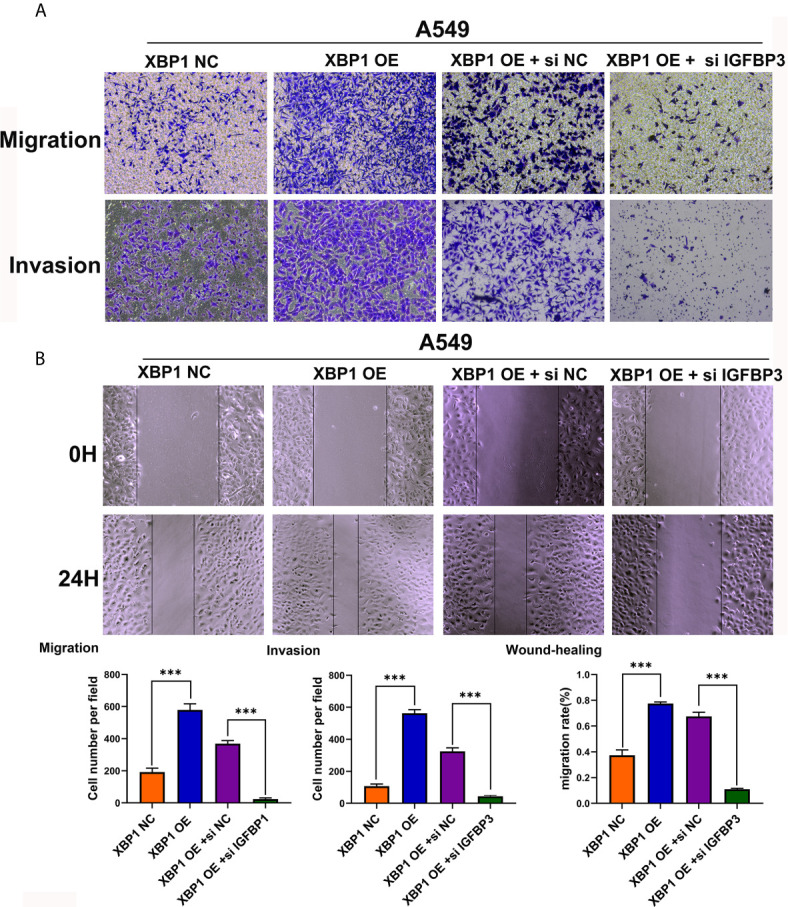
IGFBP3 downregulation alleviates XBP1 induced lung cancer cells’ migratory capacity and invasive ability *in vitro*. **(A)** Representative images and quantification of the transwell migration and invasion assay using transfected A549 cells. **(B)** Representative images and quantification of the wound-healing assay using transfected A549 cells. Data were represented as the mean ± SEM of three independent experiments. ***P < 0.001.

After observing the interaction between XBP1 and IGFBP3, we then investigated whether XBP1 regulates NSCLC cells invasion and metastasis by regulating IGFBP3, we transfected IGFBP3 siRNA into A549 cells for further experiments (**Figure 5E**). The results of the wound-healing assay (**Figure 6B**) and the transwell invasion assay and metastasis assay (**Figure 6A**) indicated si-IGFBP3 inhibits the effects of XBP1 in NSCLC cells migration, invasion and metastasis. Research has reported that MMPs is involved in XBP1-regulated cancer progress in kinds of cancers (16, 21). we hypothesized XBP1/IGFBP3/MMP-9 axis can regulates NSCLC cells invasion and metastasis. We then examined protein levels of MMP-9 using Western blot, their alteration was consistent with the findings from these vitro experiments (**Figure 5F**). Taken together, our data suggest that XBP1 regulates NSCLC cells invasion and metastasis by regulating IGFBP3/MMP-9 axis.

## Discussion

Lung cancer is the leading cause of cancer death worldwide (1). Tumor metastasis and recurrence are the main reasons for the poor prognosis of patients with NSCLC, but which drive molecules in these processes and the molecular mechanisms still not clear. In this study, we identified a novel role of XBP1 as a tumor promoter in the tumorigenesis and tumor progression of NSCLC. First, we explored the expression and clinical significance of XBP1s protein in NSCLC. we confirmed that XBP1 was significantly increased in NSCLC tumor tissue and cell lines, and overexpresses XBP1 was associated with TNM stages and lymph node metastasis in NSCLC and the expression of XBP1 was related with the 3-year OS. Second, we further explored the ability of XBP1 in regulating the migration and invasion of NSCLC cells. The transwell assay and wound healing assay showed that overexpressed XBP1 can significantly promotes NSCLC cell migration, invasion and metastasis *in vitro*. Moreover, the further experiments showed XBP1 promotes NSCLC proliferation and metastasis *in vivo*. Our data provided the compelling evidence that XBP1 functions as regulator promotes NSCLC cell invasion and metastasis in NSCLC progression.

XBP1 has been reported be a potent oncogenic protein in the process of tumorigenesis and metastasis in various cancers *via* various signal pathway. Interestingly, it has been reported that XBP1 upregulates antioxidant molecule catalase to protect against oxidative stress (8). And downregulation of XBP1 increased the sensitivity of SOC(serous ovarian cancer) cells by increasing ROS generation after H(2)O(2) exposure (9). In triple-negative breast cancer, XBP1 has been reported to be a pivotal role in the tumorigenicity and progression and XBP1 gene expression was highly associated with HIF1α and hypoxia-driven signatures (18). Li et al. revealed that overexpression of XBP1 mediates the progression of breast cancer through regulating the key EMT regulator Snail (17). In 2018, Sun et al. found the expression of XBP1 is significantly increased in oral squamous cell carcinoma (OSCC) with lymph nodes metastasis and regulates tumor invasion and poor prognosis in OSCC *via* AXL signaling (16). In addition, XBP1s can directly bind to the tumor suppressor TAp73 promoter to suppress its expression, the further experiments showed that Tap73 plays a critical role in XBP1s-induced tumorigenesis (20). However, the mechanisms by which XBP1 affects migration capacity and metastatic ability in NSCLC remains unknown. To explore the possible downstream molecular factor of XBP1 involved in NSCLC metastasis, we performed mass spectrometry analysis in A549 ^XBP1-OE^ and A549 ^XBP1-NC^ cells, and found that IGFBP3 protein can interact with XBP1. Insulin-like growth factor binding protein-3 (IGFBP3) is the primary carrier of insulin growth factors (IGFs) and also a major regulator of growth hormone secretion and IGF action (24, 25). Previous studies have shown that IGFBP3 plays a crucial role in the tumor progression in various cancers, such as colorectal cancer (26), squamous esophageal cancer (27), nasopharyngeal carcinoma (28), glioma (29), and lung adenocarcinoma (30). In addition, it has been reported that up-regulated of IGFBP3 promoted A549 cell migration and invasion, and further analysis showed overexpression of IGFBP3 might mediate brain metastasis in lung adenocarcinoma, these data indicated IGFBP3 was associated with tumorigenesis and development in lung adenocarcinoma (30). In this study, we found that IGFBP3 levels are significantly upregulated in NSCLC tissues by analyzing available ONCOMINE database and qRT-PCR. And we confirmed that the expression of XBP1 effectively promotes the expression of IGFBP3 by using Western blot. Moreover, Co-immunoprecipitation showed the combination between XBP1 and IGFBP3. After finding that XBP1 regulates the expression of IGFBP3, we further explored whether IGFBP3 downregulation can alleviate the NSCLC migratory and metastasis ability that XBP1 induced. Here, our results suggest that XBP1 can regulate the expression of IGFBP3 to regulate NSCLC migration and invasion. Moreover, matrix metalloproteinases (MMPs) are the most prominent proteinases family associated with tumorigenesis. And MMPs play an essential role in the complex systems that regulate tumor invasion and metastasis. Secreted proteases, such as MMP-2, MMP-9 promote tumor cell invasion and metastasis by digest the ECM and cell adhesion proteins (31). In previous study, they revealed that XBP1 promotes the development of ESCC by activating MMP-9 expression (21). And we hypothesized MMP-9 may play an important role in the process of XBP1 regulating NSCLC tumorigenesis and metastasis. Further Western blot assay showed that overexpressed XBP1 can upregulate the expression levels of MMP-9 and IGFBP3 downregulation can alleviate high expression of MMP-9 that XBP1 induced. However, the specific mechanism of XBP1 affecting IGFBP3 expression in NSCLC remains unclear, there are studies showed that activation of XBP1 can induce the expression of EMT-associated transcription factors (TFs), including ZEB2, TCF3 and snail1 and other EMT-associated molecule such as TGF-β (7, 11, 17). Moreover, Yang et al. found that TGF-β can elevate expression levels of IGFBP3 (30). We hypothesized XBP1 may affect IGFBP3 expression by regulating the expression of EMT-TFs and other molecules. All of them need further investigation.

In summary, our current work revealed XBP1 is a novel biomarker of NSCLC, which was closely associated with tumor lymph node metastasis, advanced clinical stages and poor prognosis. We demonstrated a novel mechanism of tumor invasion and metastasis in NSCLC that involves the activation of the XBP1/IGFBP3/MMP-9 pathway. Collectively, XBP1 not only has a critical role in NSCLC progression but also be a potential therapeutic targets and biomarker in NSCLC.

## Data Availability Statement

The original contributions presented in the study are included in the article/**Supplementary Material**, further inquiries can be directed to the corresponding author.

## Ethics Statement

The studies involving human participants were reviewed and approved by Ethics Committee of Xiangya Hospital, Central South University Approval documents for scientific research projects. The patients/participants provided their written informed consent to participate in this study. The animal study was reviewed and approved by Ethics Committee of Xiangya Hospital, Central South University Approval documents for scientific research projects.

## Author Contributions

Conceived and designed the experiments: QL, WS, and CD. Perform the experiments: QL, WS, JW, WP, and XiaL. Formal analysis and Data curation: DZ, FT, XizL, JL, and SW. Writing—original draft preparation and Writing—review and editing: QL and CD. All authors contributed to the article and approved the submitted version.

## Funding 

This work was supported by the Natural Science Foundation of China (Nos: 81974367, 82003065, 81572281, 81372515), Natural Science Foundation of Hunan Province(2018JJ6131) and National Multidisciplinary Cooperative Diagnosis and Treatment Capacity Building Project for Major Diseases (Lung Cancer).

## Conflict of Interest

The authors declare that the research was conducted in the absence of any commercial or financial relationships that could be construed as a potential conflict of interest.

## Supplementary Material

Supplementary Figure 1 The expression of the selected 7 genes (IGFBP3, USP47, SEC24D, PYCR1, MANF, FKBP2, DNAJB2) in NSCLC to the normal lung tissues by analyzing available ONCOMINE database.

## Abbreviations

XBP1, X-box binding protein 1; UPR, unfolded protein response; ER, endoplasmic reticulum; NSCLC, non-small cell lung cancer; ANLT, adjacent normal lung tissues; IHC, Immunohistochemical; IGFBP3, insulin-like growth factor binding protein-3; XBP1s, spliced XBP1; XBP1u, un-spliced XBP1; IRR1α-XBP1, Inositol requiring enzyme 1α; OSCC, oral squamous cell carcinoma; STR, short tandem repeats; SDS-PAGE, sodium dodecyl sulfate polyacrylamide gel electrophoresis; OS, overall survival; IGFs, insulin growth factors.

## References

[1] Siegel RL Miller KD Jemal A. Cancer Statistics, 2020. CA Cancer J Clin (2020) 70:7–30. 10.3322/caac.21590 31912902

[2] Zhang W Cai X Yu J Lu X Qian Q Qian W. Exosome-Mediated Transfer of lncRna RP11838N2.4 Promotes Erlotinib Resistance in non-Small Cell Lung Cancer. Int J Oncol (2018) 53:527–38. 10.3892/ijo.2018.4412 PMC6017264 29845246

[3] Siegel RL Miller KD Jemal A. Cancer Statistics, 2019. CA Cancer J Clin (2019) 69:7–34. 10.3322/caac.21551 30620402

[4] Osmani L Askin F Gabrielson E Li QK. Current WHO Guidelines and the Critical Role of Immunohistochemical Markers in the Subclassification of non-Small Cell Lung Carcinoma (NSCLC): Moving From Targeted Therapy to Immunotherapy. Semin Cancer Biol (2018) 52:103–9. 10.1016/j.semcancer.2017.11.019 PMC5970946 29183778

[5] Jonna S Subramaniam DS. Molecular Diagnostics and Targeted Therapies in non-Small Cell Lung Cancer (NSCLC): An Update. Discov Med (2019) 27:167–70. 31095926

[6] Shi W Chen Z Li L Liu H Zhang R Cheng Q. Unravel the Molecular Mechanism of XBP1 in Regulating the Biology of Cancer Cells. J Cancer (2019) 10:2035–46. 10.7150/jca.29421 PMC6548171 31205564

[7] Chen S Chen J Hua X Sun Y Cui R Sha J. The Emerging Role of XBP1 in Cancer. BioMed Pharmacother (2020) 127:110069. 10.1016/j.biopha.2020.110069 32294597

[8] Liu Y Zhang X Liang Y Yu H Chen X Zheng T. Targeting X Box-Binding Protein-1 (XBP1) Enhances Sensitivity of Glioma Cells to Oxidative Stress. Neuropathol Appl Neurobiol (2011) 37:395–405. 10.1111/j.1365-2990.2010.01155.x 21138464

[9] Zhang GH Kai JY Chen MM Ma Q Zhong AL Xie SH. Downregulation of XBP1 Decreases Serous Ovarian Cancer Cell Viability and Enhances Sensitivity to Oxidative Stress by Increasing Intracellular ROS Levels. Oncol Lett (2019) 18:4194–202. 10.3892/ol.2019.10772 PMC6757316 31579421

[10] Shen X Zhang K Kaufman RJ. The Unfolded Protein Response–a Stress Signaling Pathway of the Endoplasmic Reticulum. J Chem Neuroanat (2004) 28:79–92. 10.1016/j.jchemneu.2004.02.006 15363493

[11] Jin C Jin Z Chen NZ Lu M Liu CB Hu WL. Activation of IRE1α-XBP1 Pathway Induces Cell Proliferation and Invasion in Colorectal Carcinoma. Biochem Biophys Res Commun (2016) 470:75–81. 10.1016/j.bbrc.2015.12.119 26742428

[12] Chen C Zhang X. IRE1α-XBP1 Pathway Promotes Melanoma Progression by Regulating IL-6/STAT3 Signaling. J Transl Med (2017) 15:42. 10.1186/s12967-017-1147-2 28222747 PMC5320675

[13] Fang P Xiang L Huang S Jin L Zhou G Zhuge L. IRE1α-XBP1 Signaling Pathway Regulates IL-6 Expression and Promotes Progression of Hepatocellular Carcinoma. Oncol Lett (2018) 16:4729–36. 10.3892/ol.2018.9176 PMC6126152 30214606

[14] Sheng X Nenseth HZ Qu S Kuzu OF Frahnow T Simon L. IRE1α-XBP1s Pathway Promotes Prostate Cancer by Activating c-MYC Signaling. Nat Commun (2019) 10:323. 10.1038/s41467-018-08152-3 30679434 PMC6345973

[15] Yang J Cheng D Zhou S Zhu B Hu T Yang Q. Overexpression of X-Box Binding Protein 1 (XBP1) Correlates to Poor Prognosis and Up-Regulation of PI3K/mTOR in Human Osteosarcoma. Int J Mol Sci (2015) 16:28635–46. 10.3390/ijms161226123 PMC4691070 26633383

[16] Sun Y Jiang F Pan Y Chen X Chen J Wang Y. XBP1 Promotes Tumor Invasion and is Associated With Poor Prognosis in Oral Squamous Cell Carcinoma. Oncol Rep (2018) 40:988–98. 10.3892/or.2018.6498 29916547

[17] Li H Chen X Gao Y Wu J Zeng F Song F. XBP1 Induces Snail Expression to Promote Epithelial- to-Mesenchymal Transition and Invasion of Breast Cancer Cells. Cell Signal (2015) 27:82–9. 10.1016/j.cellsig.2014.09.018 25280941

[18] Chen X Iliopoulos D Zhang Q Tang Q Greenblatt MB Hatziapostolou M. XBP1 Promotes Triple-Negative Breast Cancer by Controlling the HIF1α Pathway. Nature (2014) 508:103–7. 10.1038/nature13119 PMC4105133 24670641

[19] Tavernier Q Legras A Didelot A Normand C Gibault L Badoual C. High Expression of Spliced X-Box Binding Protein 1 in Lung Tumors is Associated With Cancer Aggressiveness and Epithelial-to-Mesenchymal Transition. Sci Rep (2020) 10:10188. 10.1038/s41598-020-67243-8 32576923 PMC7311525

[20] Ji H Huang C Wu S Kasim V. XBP1-s Promotes Colorectal Cancer Cell Proliferation by Inhibiting TAp73 Transcriptional Activity. Biochem Biophys Res Commun (2019) 508:203–9. 10.1016/j.bbrc.2018.11.112 30473215

[21] Xia T Tong S Fan K Zhai W Fang B Wang SH. XBP1 Induces MMP-9 Expression to Promote Proliferation and Invasion in Human Esophageal Squamous Cell Carcinoma. Am J Cancer Res (2016) 6:2031–40. PMC5043112 27725908

[22] Peng Z Wang J Shan B Li B Peng W Dong Y. The Long Noncoding RNA LINC00312 Induces Lung Adenocarcinoma Migration and Vasculogenic Mimicry Through Directly Binding YBX1. Mol Cancer (2018) 17:167. 10.1186/s12943-018-0920-z 30470227 PMC6260658

[23] Peng W He D Shan B Wang J Shi W Zhao W. LINC81507 Act as a Competing Endogenous RNA of miR-199b-5p to Facilitate NSCLC Proliferation and Metastasis Via Regulating the CAV1/STAT3 Pathway. Cell Death Dis (2019) 10:533. 10.1038/s41419-019-1740-9 31296840 PMC6624296

[24] Ranke MB Elmlinger M. Functional Role of Insulin-Like Growth Factor Binding Proteins. Horm Res (1997) 48 Suppl 4:9–15. 10.1159/000191304 9350439

[25] Jogie-Brahim S Feldman D Oh Y. Unraveling Insulin-Like Growth Factor Binding Protein-3 Actions in Human Disease. Endocr Rev (2009) 30:417–37. 10.1210/er.2008-0028 PMC2819737 19477944

[26] Chan YX Alfonso H Paul Chubb SA Ho KKY Gerard Fegan P Hankey GJ. Higher IGFBP3 is Associated With Increased Incidence of Colorectal Cancer in Older Men Independently of IGF1. Clin Endocrinol (Oxf) (2018) 88:333–40. 10.1111/cen.13499 29044573

[27] Yoshino K Motoyama S Koyota S Shibuya K Usami S Maruyama K. IGFBP3 and BAG1 Enhance Radiation-Induced Apoptosis in Squamous Esophageal Cancer Cells. Biochem Biophys Res Commun (2011) 404:1070–5. 10.1016/j.bbrc.2010.12.115 21195059

[28] Bao L Liu H You B Gu M Shi S Shan Y. Overexpression of IGFBP3 is Associated With Poor Prognosis and Tumor Metastasis in Nasopharyngeal Carcinoma. Tumour Biol (2016) 37:15043–52. 10.1007/s13277-016-5400-8 27658775

[29] Chen CH Chen PY Lin YY Feng LY Chen SH Chen CY. Suppression of Tumor Growth Via IGFBP3 Depletion as a Potential Treatment in Glioma. J Neurosurg (2019) 132:168–79. 10.3171/2018.8.Jns181217 30641835

[30] Yang L Li J Fu S Ren P Tang J Wang N. Up-Regulation of Insulin-like Growth Factor Binding Protein-3 is Associated With Brain Metastasis in Lung Adenocarcinoma. Mol Cells (2019) 42:321–32. 10.14348/molcells.2019.2441 PMC6530643 31085806

[31] Forsyth PA Wong H Laing TD Rewcastle NB Morris DG Muzik H. Gelatinase-a (MMP-2), Gelatinase-B (MMP-9) and Membrane Type Matrix Metalloproteinase-1 (MT1-MMP) are Involved in Different Aspects of the Pathophysiology of Malignant Gliomas. Br J Cancer (1999) 79:1828–35. 10.1038/sj.bjc.6690291 PMC2362801 10206300

