# Supplementary figures and images for: XBP1- IGFBP3 Signaling Pathway Promotes NSCLC Invasion and Metastasis
Source: Front Oncol. 2021 May 18;11:654995. doi: 10.3389/fonc.2021.654995 (PMC8169999; doi:10.3389/fonc.2021.654995)

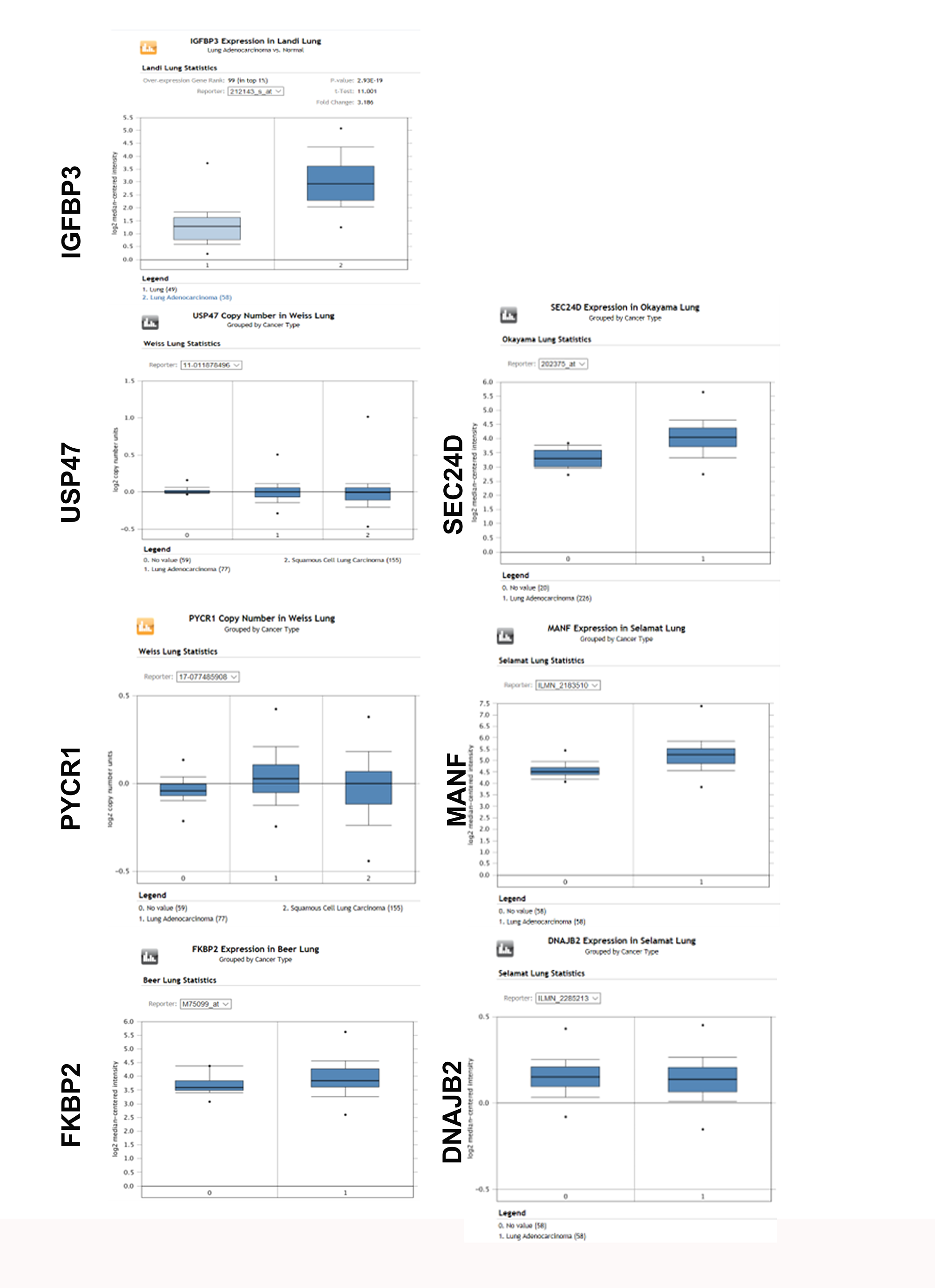

Supplement: Supplementary Figure 1 — The expression of the selected 7 genes (IGFBP3, USP47, SEC24D, PYCR1, MANF, FKBP2, DNAJB2) in NSCLC to the normal lung tissues by analyzing available ONCOMINE database. [file Image_1.tif]
